# Supplementary material for: Temporal trends of severity and outcomes of critically ill patients with COVID-19 after the emergence of variants of concern: A comparison of two waves
Source: PLoS One. 2024 Mar 7;19(3):e0299607. doi: 10.1371/journal.pone.0299607 (PMC10919739; doi:10.1371/journal.pone.0299607)
Supplement: S1 Text — (DOCX) [file pone.0299607.s001.docx]

## **S1 Text. Supplementary Methods**

## **Diagnosis of COVID-19**

The diagnosis of COVID-19 was based on clinical history, typical radiological findings of COVID-19 plus laboratorial confirmation. Positive results on either Reverse Transcription Polymerase Chain Reaction (RT-PCR), antigen test or antibody (serology) test were used to confirm SARS-CoV-2 infection.

Respiratory specimens (nasal or throat swabs specimens or tracheal aspirate) were collected at hospital admission, but some patients who were transferred from other health service had already arrived at the hospital with confirmed laboratory diagnosis. If RT-PCR or antigen test was negative, but there was a high clinical suspicion of COVID-19, the test was repeated or a blood sample was obtained for a quantitative antibody test for IgM and IgG, which was performed after seven days of the onset of symptoms and, if necessary, repeated after at least 14 days after the first blood sample.

## **Detection of SARS-CoV-2 variants**

Patients admitted in the second wave were screened for infection by SARS-CoV-2 variants. Testing was performed for all patients who had a respiratory sample available for genetic testing. We tested samples from 67 (25%) of the 268 patients, since many samples were unavailable, either because RT-PCR was done in another health service, or because COVID-19 diagnosis was made using antigen or antibody tests. Nasal swab and tracheal aspirate samples were tested for detection of SARS-CoV-2 variants. We used a protocol where through specific mutations of each variant it is possible to identify them. Using the TaqPathTM 1-Step RT-qPCR Master Mix (Thermo Fisher Scientific, Waltham, MA, USA) CG (4X), TaqManTM SARS-CoV-2 Mutation Panel Assay (40X) (Thermo Fisher Scientific) and NFW, the RT- qPCR was prepared. Samples were tested for each mutation (Panel 1) using the QuantStudio™ 5 Real-Time PCR System (Applied Biosystem, Foster City, California, USA). The variant was identified according to the positivity for each mutation. We detected Gamma Variant in 63 (94%) of the samples, Alpha Variant in one sample and four samples had indeterminate lineage.

**Panel 1. SARS-CoV-2 Variant Panel – Thermo Fisher**


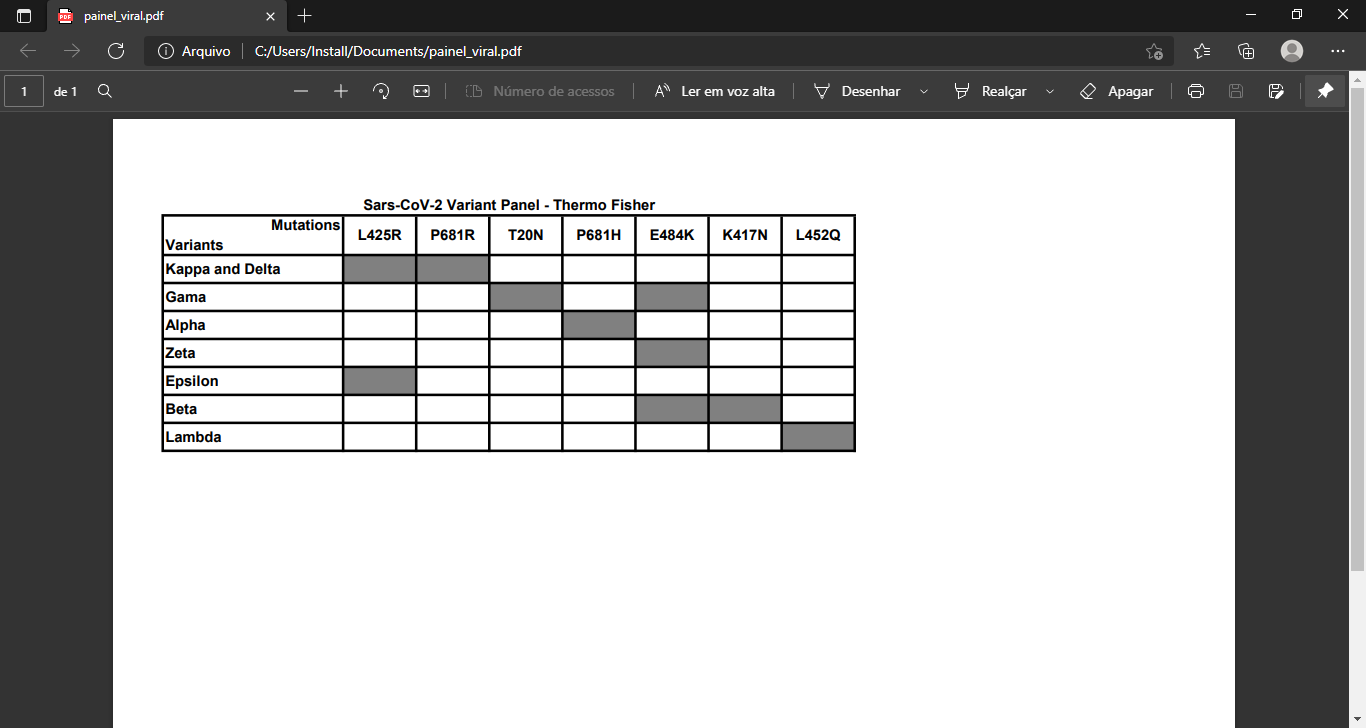


**L452R**

## Supplementary Tables

## 
