# Supplementary figures and images for: Temporal trends of severity and outcomes of critically ill patients with COVID-19 after the emergence of variants of concern: A comparison of two waves
Source: PLoS One. 2024 Mar 7;19(3):e0299607. doi: 10.1371/journal.pone.0299607 (PMC10919739; doi:10.1371/journal.pone.0299607)

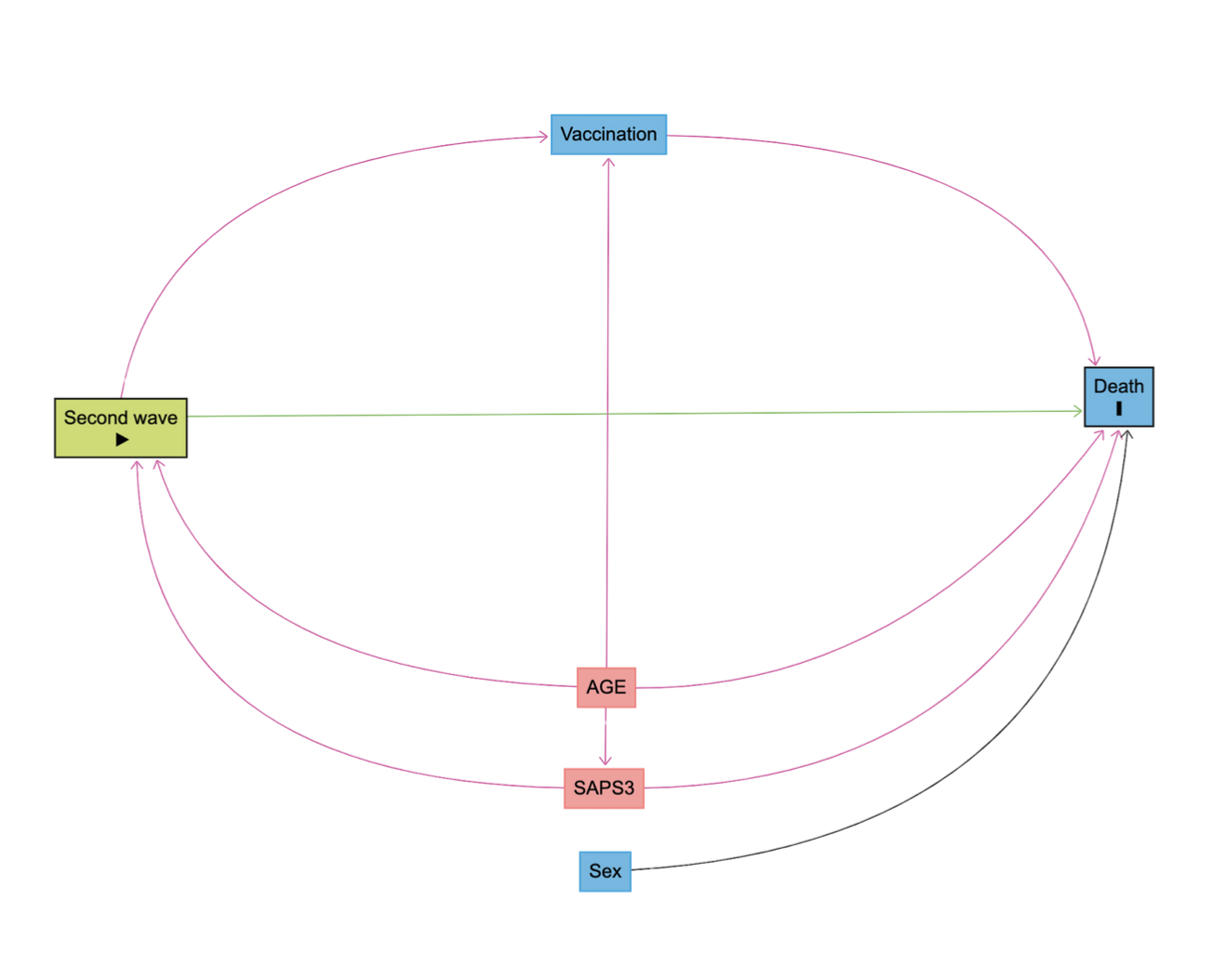

Supplement: S1 Fig — SAPS 3: Simplified acute Physiology Score 3. This conceptual model shows clinically relevant variables associated with survival. Arrows indicate a presumed direct causal effect of one variable on another variable. Admission in the second wave is the main predictor, shown in green; variables associated with the outcome, but not associated with the main predictor, are shown in blue; variables associated with both the outcome and the main predictor are shown in red (arrows indicate a suspected direct causal effect of that variable on both the main predictor and the outcome). A multivariate analysis for estimating the direct effect of admission in the second wave on survival should be adjusted for potential confounders, identified in the model as age, SAPS3 and vaccination. (TIF) [file pone.0299607.s002.tif]

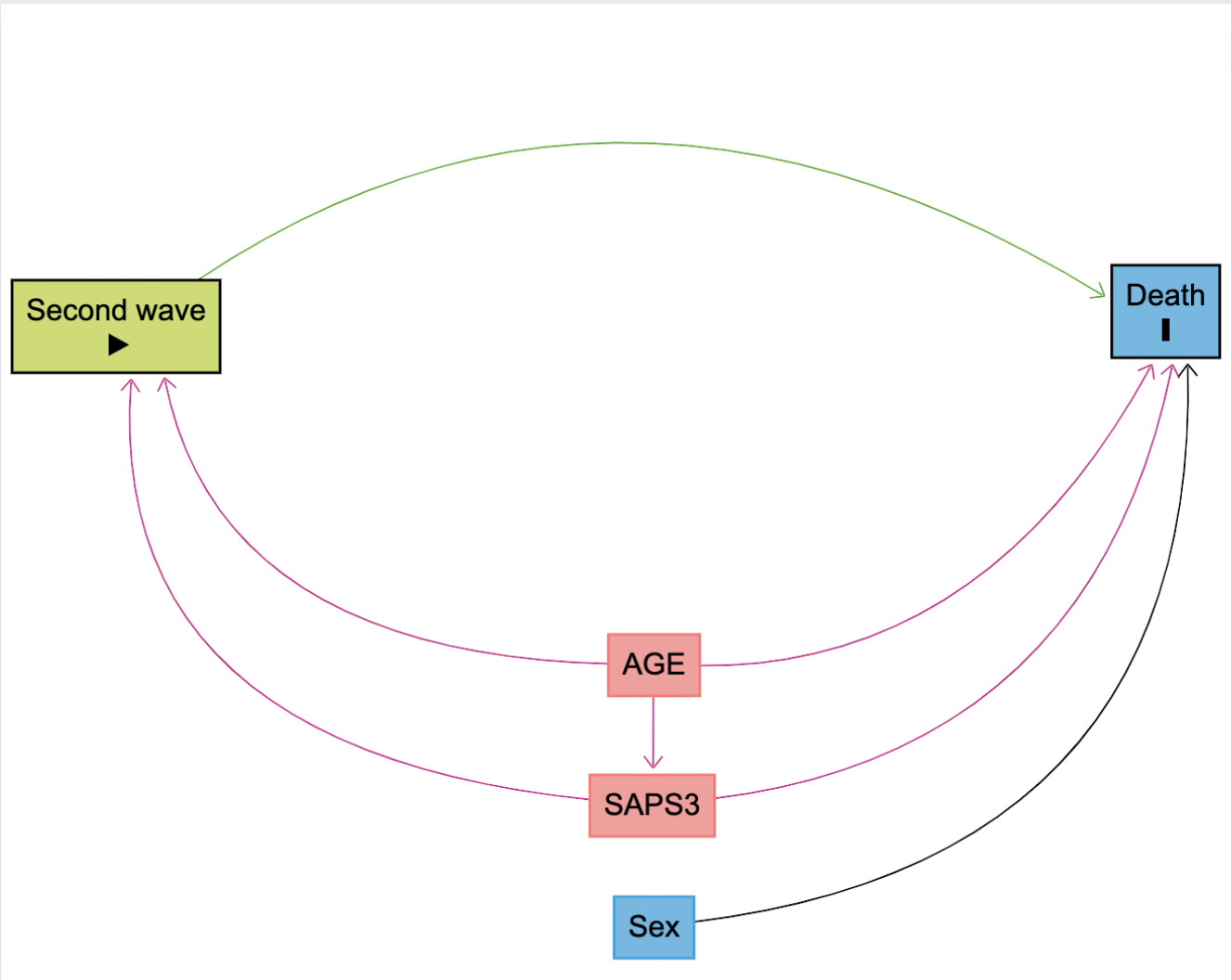

Supplement: S2 Fig — SAPS 3: Simplified acute Physiology Score 3. This conceptual model, used as a sensitivity analysis, shows clinically relevant variables associated with survival, not including vaccination. Arrows indicate a presumed direct causal effect of one variable on another variable. Admission in the second wave is the main predictor, shown in green; variables associated with the outcome, but not associated with the main predictor, are shown in blue; variables associated with both the outcome and the main predictor are shown in red (arrows indicate a suspected direct causal effect of that variable on both the main predictor and the outcome). A multivariate analysis for estimating the direct effect of admission in the second wave on survival should be adjusted for potential confounders, identified in the model as age and SAPS3. (TIF) [file pone.0299607.s003.tif]
