# Supplementary material for: Temporal trends of severity and outcomes of critically ill patients with COVID-19 after the emergence of variants of concern: A comparison of two waves
Source: PLoS One. 2024 Mar 7;19(3):e0299607. doi: 10.1371/journal.pone.0299607 (PMC10919739; doi:10.1371/journal.pone.0299607)
Supplement: S1 Table — Data are presented as median [IQR]: interquartile range; comparisons were made with Mann-Whitney test. Missing year 2020: Arterial lactate for 399 (30%) patients; D-dimer for 316 (24%) patients; Arterial pH for 151 (12%) patients; C-reactive protein for 202 (15%) patients. Missing year 2021: Arterial lactate for 65 (24%) patients; D-dimer for 12 (5%) patients; Arterial pH for 45 (17%) patients; C-reactive protein for 10 (4%) patients. (DOCX) [file pone.0299607.s004.docx]

| **S1 Table – Laboratory tests at ICU admission** |
| --- |
| \|  \| **First wave (n=1315)** \| **Second wave (n=268)** \| ***p* value** \| \| --- \| --- \| --- \| --- \| \| Arterial lactate (mg/dL) \| 15 [12 – 20] \| 16 [13 – 19] \| 0.071 \| \| Creatinine (mg/dL) \| 1.20 [0.79 - 2.32] \| 0.99 [0.76 - 1.52] \| <0.001 \| \| D-dimer (ng/dL) \| 2135 [1085 - 6856] \| 2131 [989 – 7606] \| 0.762 \| \| Arterial pH \| 7.38 [7.32 - 7.44] \| 7.39 [7.33 - 7.45] \| 0.138 \| \| White blood cell count (/mm3) \| 9650 [6840 - 13725] \| 11300 [8092 - 14610] \| <0.001 \| \| Lymphocyte count (/mm3) \| 800 [520 - 1180] \| 720 [450 – 995] \| <0.001 \| \| Platelet count (1000/mm3) \| 223 [162 - 298] \| 232 [179 - 300] \| 0.097 \| \| C-reactive protein (mg/L) \| 182 [92 - 286] \| 130 [72 – 198] \| <0.001 \| |
| Data are presented as median [IQR]: interquartile range; comparisons were made with Mann-Whitney test.  Missing year 2020: Arterial lactate for 399 (30%) patients; D-dimer for 316 (24%) patients; Arterial pH for 151 (12%) patients; C-reactive protein for 202 (15%) patients. Missing year 2021: Arterial lactate for 65 (24%) patients; D-dimer for 12 (5%) patients; Arterial pH for 45 (17%) patients; C-reactive protein for 10 (4%) patients. |
