# Supplementary material for: Temporal trends of severity and outcomes of critically ill patients with COVID-19 after the emergence of variants of concern: A comparison of two waves
Source: PLoS One. 2024 Mar 7;19(3):e0299607. doi: 10.1371/journal.pone.0299607 (PMC10919739; doi:10.1371/journal.pone.0299607)
Supplement: S2 Table — Definition of abbreviations: O2: oxygen; RASS: Richmond Agitation-Sedation Scale; COVID-19: Coronavirus Disease 2019. Data are n. (%); comparisons were made with the chi-square test. (DOCX) [file pone.0299607.s005.docx]

| **Table S2 – Patient management on the first 24 h after ICU admission** |
| --- |
| \| **Management** \| **First wave (n=1315)** \| **Second wave (n=268)** \| ***p* value** \| \| --- \| --- \| --- \| --- \| \| RASS, median [IQR] \| -5 [-4 – -5] \| -5 [-5 – -5] \| <0.001 \| \| **Sedation, n (%)** \| 834 (63) \| 146 (55) \| 0.007 \| \| Midazolam \| 709 (54) \| 122 (45) \| 0.015 \| \| Propofol \| 158 (12) \| 67 (25) \| <0.001 \| \| Fentanyl \| 730 (55) \| 126 (47) \| 0.013 \| \| ketamine \| 33 (2.5) \| 18 (7) \| 0.001 \| \| Dexmedetomidine \| 8 (0.6) \| 5 (1.9) \| 0.088 \| \| Neuromuscular blockade, n (%) \| **352 (27)** \| **105 (39)** \| <0.001 \| \| Delirium, n (%) \| 59 (4) \| 8 (3) \| 0.344 \| \| **Vasoactive drugs, n (%)** \| 550 (42) \| 80 (30) \| <0.001 \| \| Norepinephrine \| 500 (38) \| 64 (24) \| <0.001 \| \| Vasopresin \| 46 (3.5) \| 3 (2.2) \| 0.386 \| \| Adrenaline \| 3 (0.2) \| 3 (1.1) \| 0.105 \| \| Nitroglycerin \| 1 (0.1) \| 1 (0.4) \| 0.761 \| \| Nitroprusside \| 12 (0.9) \| 9 (3.4) \| 0.004 \| \| Dobutamine, n (%) \| 69 (5.2) \| 10 (3.7) \| 0.376 \| \| **Respiratory support, n (%)** \|  \|  \| <0.001 \| \| Invasive mechanical ventilation \| 878 (67) \| 148 (55) \|  \| \| High flow nasal cannula \| 35 (2.6) \| 62 (23) \|  \| \| Non-invasive ventilation \| 61 (4.6) \| 23 (8.6) \|  \| \| O2 catheter \| 124 (10) \| 19 (7) \|  \| \| Venturi mask \| 93 (7) \| 3 (1) \|  \| \| Nonrebreathing mask \| 71 (5.4) \| 13 (5) \|  \| \| Room air \| 53 (4.0) \| 0 (0.0) \|  \| \| Renal Replacement Therapy, n (%) \| 104 (8) \| 16 (6) \| 0.334 \| \| Antibiotics, n (%) \| 906 (69) \| 113 (42) \| <0.001 \| \| **COVID-19 specific treatment, n (%)** \|  \|  \|  \| \| Corticosteroids \| 324 (25) \| 264 (98) \| <0.001 \| \| Tocilizumab \| 3 (0.2) \| 12 (4.5) \| <0.001 \| \| **Anticoagulation, n (%)** \|  \|  \| <0.001 \| \| None \| 115 (9) \| 14 (5) \|  \| \| Prophylactic dose \| 1077(82) \| 204 (76) \|  \| \| Therapeutic dose \| 123 (9) \| 50 (19) \|  \| |
| Definition of abbreviations: O_2_: oxygen; RASS: Richmond Agitation-Sedation Scale; COVID-19: Coronavirus Disease 2019. Data are n. (%); comparisons were made with the chi-square test. |
