# Supplementary material for: Temporal trends of severity and outcomes of critically ill patients with COVID-19 after the emergence of variants of concern: A comparison of two waves
Source: PLoS One. 2024 Mar 7;19(3):e0299607. doi: 10.1371/journal.pone.0299607 (PMC10919739; doi:10.1371/journal.pone.0299607)
Supplement: S4 Table — HR: hazard ratio; aHR: adjusted hazard ratio; 95%CI: 95% confidence interval; SAPS 3: Simplified acute Physiology Score 3; HR, aHR obtained with univariate and multivariate Cox models, respectively, and 95%CI and p values obtained in each model. SAPS 3 was missing for 1 patient. (DOCX) [file pone.0299607.s007.docx]

| **S4 Table – Sensitivity analysis - Association between admission in the second wave, other relevant covariates, and 60-day survival** |
| --- |
| \| **Characteristic** \| **HR** \| ***p* value** \| **aHR** \| ***p* value** \| \| --- \| --- \| --- \| --- \| --- \| \| Admission in the 2º wave \| 0.61 (95%CI 0.50 – 0.76) \| <0.001 \| 0.89 (95%CI 0.72 – 1.12) \| 0.339 \| \| Age \| 1.03 (95%CI 1.03 – 1.04) \| <0.001 \| 1.02 (95%CI 1.02 – 1.03) \| <0.001 \| \| SAPS 3 \| 1.04 (95%CI 1.04 – 1.05) \| <0.001 \| 1.03 (95%CI 1.03 – 1.04) \| <0.001 \| |
| HR: hazard ratio; aHR: adjusted hazard ratio; 95%CI: 95% confidence interval; SAPS 3: Simplified acute Physiology Score 3; HR, aHR obtained with univariate and multivariate Cox models, respectively, and 95%CI and *p* values obtained in each model. SAPS 3 was missing for 1 patient. |
