# Supplementary material for: Temporal trends of severity and outcomes of critically ill patients with COVID-19 after the emergence of variants of concern: A comparison of two waves
Source: PLoS One. 2024 Mar 7;19(3):e0299607. doi: 10.1371/journal.pone.0299607 (PMC10919739; doi:10.1371/journal.pone.0299607)
Supplement: Attachment — Submitted filename: PONE-D-23-21314_reviewer.pdf [file pone.0299607.s008.pdf]

PLOS ONE

TEMPORAL TRENDS OF SEVERITY AND OUTCOMES OF CRITICALLY ILL PATIENTS WITH COVID-19 AFTER THE EMERGENCE OF VARIANTS OF CONCERN: A COMPARISON OF TWO COHORTS

--Manuscript Draft--

**Manuscript Number:**	PONE-D-23-21314
**Article Type:**	Research Article
**Full Title:**	TEMPORAL TRENDS OF SEVERITY AND OUTCOMES OF CRITICALLY ILL PATIENTS WITH COVID-19 AFTER THE EMERGENCE OF VARIANTS OF CONCERN: A COMPARISON OF TWO COHORTS
**Short Title:**	TEMPORAL TRENDS OF COVID-19 SEVERITY AFTER THE EMERGENCE OF VARIANTS OF CONCERN
**Corresponding Author:**	Daniela Helena Machado Freitas, M.D.<br>University of Sao Paulo: Universidade de Sao Paulo<br>SÃO PAULO, São Paulo BRAZIL
**Keywords:**	COVID-19; SARS-CoV-2; Acute respiratory distress syndrome; Intensive care units; Healthcare disparities
**Abstract:**	Background<br><br>The emergence of SARS-CoV-2 variants led to subsequent waves of COVID-19 worldwide. In many countries, the second wave of COVID-19 was marked by an overflow of severe cases and record deaths, raising the concern that variants associated with that wave might be more deadly. Our aim was to compare outcomes of critically-ill patients of the first two waves of COVID-19.<br><br>Methods<br><br>This retrospective cohort included critically-ill patients admitted between March-June 2020 and April-July 2021 in the largest academic hospital in Brazil. We compared admission characteristics and hospital outcomes. The main outcome was 60-day survival and Cox models were used to test the association between COVID-19 wave, relevant covariates and survival.<br><br>Results<br><br>We included 1583 patients (1315 in the first and 268 in the second wave). Patients in the second wave were younger, had lower severity scores, used prone and non-invasive ventilatory support more often, and fewer patients required mechanical ventilation (70% vs 80%, p<0.001), vasopressors (60 vs 74%, p<0.001), and dialysis (22% vs 37%, p<0.001). Survival was higher in the second wave (HR 0.61, 95%CI 0.50–0.76). In the multivariable model, admission during the second wave, adjusted for age, SAPS3 and vaccination, was not associated with survival (aHR 0.85, 95%CI 0.65–1.12).<br><br>Conclusions<br><br>In this cohort study, patients with COVID-19 admitted to the ICU in the second wave were younger and had better prognostic scores. Adjusted survival was similar in the two waves, contrasting with record number of hospitalizations, daily deaths and health system collapse seen across the country in the second wave. Our findings suggest that the combination of the burden of severe cases and factors such as resource allocation and health disparities may have had an impact in the excess mortality found in many countries in the second wave.
**Order of Authors:**	Daniela Helena Machado Freitas, M.D.
	Eduardo Leite Vieira Costa, M.D.
	Natalia Alcantara Zimmermann, M.D.

Powered by Editorial Manager® and ProduXion Manager® from Aries Systems Corporation

	Larissa Santos Oliveira Gois
	Mirella Vittig Alves Anjos
	Felipe Gallego Lima, M.D.
	Pâmela Santos Andrade
	Daniel Joelsons, M.D.
	Yeh-Li Ho, M.D.
	Flávia Cristina Silva Sales
	Ester Cerdeira Sabino, M.D.
	Carlos Roberto Ribeiro Carvalho, M.D.
	Juliana Carvalho Ferreira, M.D.
**Additional Information:**	
**Question**	**Response**
**Financial Disclosure**<br><br>Enter a financial disclosure statement that describes the sources of funding for the work included in this submission. Review the submission guidelines for detailed requirements. View published research articles from *PLOS ONE* for specific examples.<br><br>This statement is required for submission and **will appear in the published article** if the submission is accepted. Please make sure it is accurate.<br><br>**Unfunded studies**<br>Enter: *The author(s) received no specific funding for this work.*<br><br>**Funded studies**<br>Enter a statement with the following details:<br>• Initials of the authors who received each award<br>• Grant numbers awarded to each author<br>• The full name of each funder<br>• URL of each funder website<br>• Did the sponsors or funders play any role in the study design, data collection and analysis, decision to publish, or preparation of the manuscript?<br>• **NO** - Include this sentence at the end of your statement: *The funders had no role in study design, data collection and analysis, decision to publish, or preparation of the manuscript.*<br>• **YES** - Specify the role(s) played.	The author(s) received no specific funding for this work.

* typeset	
**Competing Interests**<br><br>Use the instructions below to enter a competing interest statement for this submission. On behalf of all authors, disclose any competing interests that could be perceived to bias this work—acknowledging all financial support and any other relevant financial or non-financial competing interests.<br><br>This statement is required for submission and **will appear in the published article** if the submission is accepted. Please make sure it is accurate and that any funding sources listed in your Funding Information later in the submission form are also declared in your Financial Disclosure statement.<br><br>View published research articles from *PLOS ONE* for specific examples.<br><br>**NO authors have competing interests**<br><br>Enter: *The authors have declared that no competing interests exist.*<br><br>**Authors with competing interests**<br><br>Enter competing interest details beginning with this statement:<br><br>*I have read the journal's policy and the authors of this manuscript have the following competing interests: [insert competing interests here]*<br><br>* typeset	Dr. Ferreira reports personal fees from Medtronic, outside the submitted work; Dr. Costa reports personal fees from Timpel, personal fees from Magnamed, outside the submitted work; Dr. Ho reports personal fees from Pan-American Health Organization, outside the submitted work. The other authors have no conflict of interest to disclose.
**Ethics Statement**<br><br>Enter an ethics statement for this submission. This statement is required if the study involved:<br><br>• Human participants<br>• Human specimens or tissue	The Research Ethics Committee approved the study (number CAAE: 50340521.6.0000.0068). Requirement for informed consent form was waived because of the observational nature of the study.

Powered by Editorial Manager® and ProduXion Manager® from Aries Systems Corporation

• Vertebrate animals or cephalopods<br>• Vertebrate embryos or tissues<br>• Field research<br><br>Write "N/A" if the submission does not require an ethics statement.<br><br>General guidance is provided below. Consult the submission guidelines for detailed instructions. **Make sure that all information entered here is included in the Methods section of the manuscript.**	

Powered by Editorial Manager® and ProduXion Manager® from Aries Systems Corporation

**Format for specific study types**<br><br>**Human Subject Research (involving human participants and/or tissue)**<br>• Give the name of the institutional review board or ethics committee that approved the study<br>• Include the approval number and/or a statement indicating approval of this research<br>• Indicate the form of consent obtained (written/oral) or the reason that consent was not obtained (e.g. the data were analyzed anonymously)<br><br>**Animal Research (involving vertebrate animals, embryos or tissues)**<br>• Provide the name of the Institutional Animal Care and Use Committee (IACUC) or other relevant ethics board that reviewed the study protocol, and indicate whether they approved this research or granted a formal waiver of ethical approval<br>• Include an approval number if one was obtained<br>• If the study involved *non-human primates*, add *additional details* about animal welfare and steps taken to ameliorate suffering<br>• If anesthesia, euthanasia, or any kind of animal sacrifice is part of the study, include briefly which substances and/or methods were applied<br><br>**Field Research**<br><br>Include the following details if this study involves the collection of plant, animal, or other materials from a natural setting:<br>• Field permit number<br>• Name of the institution or relevant body that granted permission	
**Data Availability**<br><br>Authors are required to make all data underlying the findings described fully available, without restriction, and from the time of publication. PLOS allows rare exceptions to address legal and ethical concerns. See the PLOS Data Policy and FAQ for detailed information.	Yes - all data are fully available without restriction

A Data Availability Statement describing where the data can be found is required at submission. Your answers to this question constitute the Data Availability Statement and **will be published in the article**, if accepted.<br><br>**Important:** Stating 'data available on request from the author' is not sufficient. If your data are only available upon request, select 'No' for the first question and explain your exceptional situation in the text box.<br><br>Do the authors confirm that all data underlying the findings described in their manuscript are fully available without restriction?	
**Describe where the data may be found in full sentences. If you are copying our sample text, replace any instances of XXX with the appropriate details.**<br><br>• If the data are **held or will be held in a public repository**, include URLs, accession numbers or DOIs. If this information will only be available after acceptance, indicate this by ticking the box below. For example: *All XXX files are available from the XXX database (accession number(s) XXX, XXX.*).<br>• If the data are all contained **within the manuscript and/or Supporting Information files**, enter the following: *All relevant data are within the manuscript and its Supporting Information files.*<br>• If neither of these applies but you are able to provide **details of access elsewhere**, with or without limitations, please do so. For example:<br><br>*Data cannot be shared publicly because of [XXX]. Data are available from the XXX Institutional Data Access / Ethics Committee (contact via XXX) for researchers who meet the criteria for access to confidential data.*<br><br>*The data underlying the results presented in the study are available from (include the name of the third party*	Our institution has an institution-wide data management plan for COVID-19 datasets which includes making anonymized data publicly available to contribute to nationwide and international registries of COVID-19 patients according to a pre-defined schedule.

and contact information or URL).<br>• This text is appropriate if the data are owned by a third party and authors do not have permission to share the data.<br><br>* typeset	
Additional data availability information:	Tick here if the URLs/accession numbers/DOIs will be available only after acceptance of the manuscript for publication so that we can ensure their inclusion before publication.

Manuscript

Click here to access/download;Manuscript;Manuscript_pone.docx

Temporal trends of severity and outcomes of critically ill patients with COVID-19 after the emergence of variants of concern: A comparison of two cohorts

Daniela Helena Machado Freitas[1*], Eduardo Leite Vieira Costa[1], Natalia Alcantara Zimmermann[1], Larissa Santos Oliveira Gois[1], Mirella Vittig Alves Anjos[1], Felipe Gallego Lima[2], Pâmela Santos Andrade[3], Daniel Joelsons[4], Yeh-Li Ho[4], Flávia Cristina Silva Sales[4,5], Ester Cerdeira Sabino[4,5], Carlos Roberto Ribeiro Carvalho[1], Juliana Carvalho Ferreira[1]

[1]Departamento de Cardiopneumologia, Divisão de Pneumologia, Instituto Do Coração, Hospital das Clínicas HCFMUSP, Faculdade de Medicina, Universidade de São Paulo, São Paulo, Brazil.

[2]Departamento de Cardiopneumologia, Divisão de Cardiologia, Instituto Do Coração, Hospital das Clínicas HCFMUSP, Faculdade de Medicina, Universidade de São Paulo, São Paulo, Brazil.

[3]Departamento de Epidemiologia, Faculdade de Saúde Pública, Universidade de São Paulo, São Paulo, Brazil.

[4]Departamento de Moléstias Infecciosas e Parasitárias, Divisão de Clínica de Moléstias Infecciosas e Parasitárias, Hospital das Clínicas HCFMUSP, Faculdade de Medicina, Universidade de São Paulo, São Paulo, Brazil.

[5]Instituto de Medicina Tropical, Faculdade de Medicina, Universidade de São Paulo, São Paulo, Brazil.

***Corresponding author**

E-mail: daniela.f@hc.fm.usp.br (DHMF)

Abstract

Background: The emergence of SARS-CoV-2 variants led to subsequent waves of COVID-19 worldwide. In many countries, the second wave of COVID-19 was marked by an overflow of severe cases and record deaths, raising the concern that variants associated with that wave might be more deadly. Our aim was to compare outcomes of critically-ill patients of the first two waves of COVID-19.

Methods: This retrospective cohort included critically-ill patients admitted between March-June 2020 and April-July 2021 in the largest academic hospital in Brazil. We compared admission characteristics and hospital outcomes. The main outcome was 60-day survival and Cox models were used to test the association between COVID-19 wave, relevant covariates and survival.

Results: We included 1583 patients (1315 in the first and 268 in the second wave). Patients in the second wave were younger, had lower severity scores, used prone and non-invasive ventilatory support more often, and fewer patients required mechanical ventilation (70% vs 80%, $p<0.001$), vasopressors (60 vs 74%, $p<0.001$), and dialysis (22% vs 37%, $p<0.001$). Survival was higher in the second wave (HR 0.61, 95%CI 0.50–0.76). In the multivariable model, admission during the second wave, adjusted for age, SAPS3 and vaccination, was not associated with survival (aHR 0.85, 95%CI 0.65–1.12).

Conclusions: In this cohort study, patients with COVID-19 admitted to the ICU in the second wave were younger and had better prognostic scores. Adjusted survival was similar in the two waves, contrasting with record number of hospitalizations, daily deaths and health system collapse seen across the country in the second wave. Our findings suggest that the combination of the burden of severe cases and factors such as resource allocation and health disparities may have had an impact in the excess

mortality found in many countries in the second wave.

Keywords: COVID-19; SARS-CoV-2; Acute respiratory distress syndrome; Intensive care units; Healthcare disparities.

Introduction

The Coronavirus Disease 2019 (COVID-19) pandemic was the most severe public health crisis of the century. By July 2023, Brazil was the sixth country in number of confirmed cases in the world ranking, with more than 37 million cases [1], and second country in cumulative deaths, with more than 700,000 deaths registered [1]. Among critically ill patients with severe acute respiratory infection (SARI) and COVID-19-related acute respiratory distress syndrome (ARDS), mortality in the first wave was high [2], especially among patients who required mechanical ventilation [3].

As countries were recovering from the toll taken by the first COVID-19 wave, the rapid worldwide spread of SARS-CoV-2 contributed to the emergence of new genetic lineages, called "variants of concern" (VOCs) [4] which showed increased transmissibility [4–6], escaped immunity generated by vaccine or natural infection [7–9], and raised concerns about increased disease severity and mortality [10–12]. The VOCs led to the worsening of the world health scenario with subsequent waves of cases and deaths. In Brazil, the gamma variant, which originated in the Amazon region, was the driver of the second wave, which was evident by January 2021 after a period of 3 months of decreasing number of cases [1,13]. In March 2021, the Brazilian government issued a warning about the presence of Gamma (P.1), Alpha (B.1.1.7) and Beta (B.1.351) variants in Brazil [14], with a proportion of the Gamma variant reaching approximately 90% of the SARS-CoV-2 lineages circulating in Brazil during this period

[5].

During the second wave, the Brazilian health system collapsed with rapidly rising number of cases, record deaths [11,15], ICU occupancy above 90% in several large cities [11,16,17], similarly to what was seen in other parts of the world [18–21]. The causes for excessive deaths, especially among the younger patients, were not clear. One initial hypothesis was that the Gamma variant could be more virulent, causing more serious illness, including among younger patients [22–25]. Other possible explanations were lack of access to vaccination, which was restricted to older and the most vulnerable populations [26,27], strain on the health system due to increased transmissibility, limited access to intensive care beds, and failure to implement public health interventions to contain community transmission [11,28].

Currently, there are limited data comparing outcomes of critically ill patients across different waves of COVID-19 focusing on the emergence of VOCs. More data are needed to clarify the interplay between different VOCs, disease severity and availability of health care resources and its impact on mortality. Therefore, we designed a cohort study to compare characteristics, clinical management, and outcomes of critically ill patients hospitalized in the first and second waves of COVID-19 in a large academic hospital in Brazil. We hypothesized that in-hospital mortality would be greater in the second wave.

Methods

Study design and location

This was a retrospective cohort conducted at Hospital das Clínicas, a public hospital affiliated with the University of Sao Paulo Medical School, and the largest academic hospital in Brazil. We compared admission characteristics and hospital outcomes of patients admitted to ICUs dedicated exclusively to the care of COVID-19 in the first and second waves of the pandemic. During the first wave, in 2020, there were 20 COVID-19 ICUs with a total of 300 beds, of which 206 beds were operating rooms or ward beds converted into ICU beds. At that time, our hospital complex was the most important referral center for COVID-19 patients from the metropolitan region of São Paulo comprising a population comparable to countries the size of Portugal. In 2021, during the second wave, referral of severe COVID-19 cases was more organized and less concentrated in our hospital, in which five ICUs remained dedicated to COVID-19, with 58 beds.

The Research Ethics Committee approved the study (number CAAE: 50340521.6.0000.0068). Requirement for informed consent form was waived because of the observational nature of the study.

Study population

From March 30 until June 30, 2020 (first wave) and April 1st until July 31, 2021 (second wave), all consecutive patients admitted to the ICU with COVID-19 were screened. Inclusion criteria were age ≥18 years and confirmed COVID-19 with Reverse Transcription Polymerase Chain Reaction (RT-PCR) or the rapid-antigen test or antibody (serology) test for IgM and IgG. Exclusion criteria were palliative care ~~on~~ in the first 24 hours after ICU admission, less than 24 hours of ICU stay, admission to the ICU after more than 7 days of invasive ventilatory support or more than 14 days of hospitalization, and asymptomatic COVID-19, defined as patients who were admitted to ICU due to other diagnosis and without symptoms of COVID-19, despite a SARS-CoV-2 RT-PCR positive on admission screening (more details in Supporting information

file).

Patient care

Patient care was at the ICU teams' discretion. The hospital developed institutional protocols specifically for COVID-19 patients, since the beginning of the pandemic, including the best evidence that emerged related to the care of critically ill patients with COVID-19. The main treatment protocol changes between 2020 and 2021 were use of corticosteroid, and reduced restrictions for the use of noninvasive ventilation (NIV) and high-flow nasal cannula (HFNC).

Outcomes

The main outcome was 60-day hospital survival. The secondary outcomes were use of protective ventilation, rescue therapies for refractory hypoxemia, need for renal replacement therapy (RRT) and/ or vasopressors.

Data collection

Patient information was collected from electronic medical records. Data were accessed from August 19, 2021, to November 12, 2021. We used an online case report form, managed on REDCap(-Research Electronic Data Capture), an online platform, ensuring the confidentiality of the information [29]. We followed patients until hospital discharge or transfer to other health services.

We collected data related to ICU admission, management in the first 24 hours, and outcomes. These data included demographic information, comorbidities, duration of symptoms, laboratory tests, prognostic scores such as Simplified Acute Physiology Score III (SAPS 3) [30] and Sequential (Sepsis-related) Organ Failure Assessment

(SOFA) [31], specific COVID-19 treatment, need for advanced life support therapies, and ventilator parameters. We defined protective ventilation as use of tidal volume < 8 mL/Kg and plateau pressure < 30 cmH_2O. We also collected ICU and hospital outcomes and clinical complications reported during the course of illness.

Information about vaccination against SARS-CoV-2 was collected from electronic medical records or telephone call to the patient's next of kin. In early 2021, vaccines approved for use in Brazil were those produced by Sinovac/Butantan Institute, AstraZeneca/Fiocruz, Pfizer/Wyeth and Janssen [27]. In São Paulo, vaccination started in mid-January 2021 for healthcare workers and was gradually expanded with priority for the most vulnerable population, the elderly and immunosuppressed. By April 2021, residents over 63 years-old had been offered their first shot and by July 2021, the cutoff was at 28 years-old [27].

Nasal swab and tracheal aspirate samples from patients admitted in the second wave were tested for detection of SARS-CoV-2 variants, using the QuantStudio™ 5 Real-Time PCR System (Applied Biosystem, Foster City, California, USA). Variant testing could not be performed for patients when samples were unavailable, either because RT-PCR was done in another health service, or because COVID-19 diagnosis was made using antigen or serologic tests (more details in Supporting information file).

We report the results according to the recommendations the Strengthening The Reporting of Observational Studies in Epidemiology (STROBE) guidelines [32].

Statistical analysis

Continuous variables were expressed as mean and standard deviation (SD) or median and interquartile range (IQR) as appropriate and compared using the independent

samples Student's t-test or Mann-Whitney U test. Categorical variables were presented as absolute and relative frequencies and compared using the Chi-square test.

We plotted Kaplan–Meier curves to estimate 60-day survival in each of the pandemic waves. 60-day survival was defined as the time interval between ICU admission and patient death from any cause or hospital discharge. Patients discharged home or transferred to other health services were considered alive at the end of follow-up. Cox proportional hazard models were used to perform survival analysis. We built a multivariable Cox model based on a conceptual causal diagram in the format of directed acyclic graph (DAG), including the association between admission in the second wave and survival, adjusted by the most relevant variables (Supporting information file: S1 Fig).

Unadjusted and adjusted hazard ratios (HR) and 95% confidence intervals (95%CI) were used to measure the association between each variable and 60-day survival. All hypothesis tests are two-tailed and p-value < 0.05 was considered statistically significant.

The analyses were performed using the statistical software R (R Foundation for Statistical Computing Platform, version 4.2.1) [33].

Results

Study Population

We screened 1,955 patients admitted to the ICU during the study periods, and included 1,583 patients, of whom 1,315 were admitted in the first and 268 were admitted in the second wave (Fig 1).

Fig 1. Study participant flow chart.

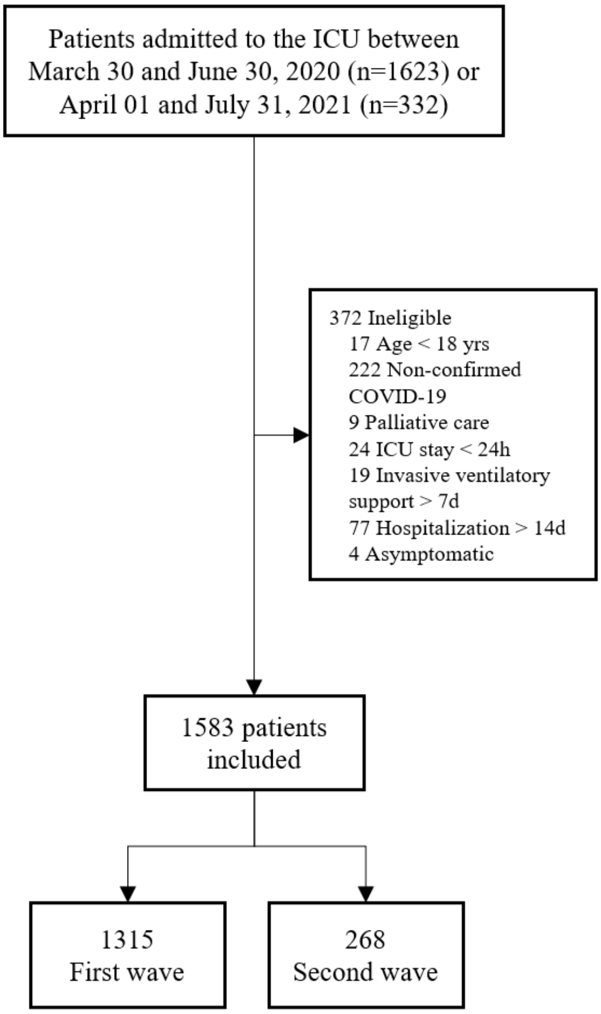

Flow of potentially eligible participants in the study, and final numbers included and analyzed
ICU: Intensive Care Unit; COVID-19: Coronavirus Disease 2019

Follow-up was complete for all patients until hospital discharge or transfer to other health services. In 2020, 104 (8%) patients were transferred to a long-term care facility, and in 2021 only 4 (1.5%) patients were transferred.

Baseline Characteristics

The baseline characteristics of the subjects, stratified by year of admission are shown in Table 1. In the first wave, patients were older (56% were ≥60 years old), than in the second wave, when 43% were ≥60 years old. Male sex was predominant in both periods. At ICU admission, patients in the first wave were more likely to be receiving mechanical ventilation and vasopressors, had more comorbidities, and the mean SAPS3 and SOFA were higher. Laboratory findings are shown in S1 Table (Supporting information file).

Table 1. Baseline characteristics at ICU admission

	First wave (n=1315)	Second wave (n=268)	*p* value
**Characteristics**			
Age (y), mean (SD)	61 ± 15	56 ± 14	<0.001
**Age groups (y), n (%)**			<0.001
18-40	138 (11)	46 (17)	
41-60	435 (33)	108 (40)	
>60	742 (56)	114 (43)	
Female sex, n (%)	520 (39)	103 (38)	0.787
BMI	28 ± 7	30 ± 6	<0.001
SAPS 3	64 ± 16	56 ± 13	<0.001
SOFA, median [IQR]	7 [3 – 10]	4 [2 – 7]	<0.001
Duration of symptoms (d), median [IQR]	9 [6 – 12]	12 [9 - 14]	<0.001
Vasopressors, n (%)	515 (39)	80 (30)	0.005
Invasive mechanical ventilation, n (%)	809 (61.5)	122 (45.5)	<0.001
Corticosteroids, n (%)	146 (11)	207 (77)	<0.001
Vaccination, n (%)	0 (0.0)	77 (29)	-
1 dose, n (%)	0 (0.0)	52 (20)	
2 doses, n (%)	0 (0.0)	25 (9)	
SARS-CoV-2 variant screening, n (%)	0 (0.0)	67 (25)	-
Gamma (P1), n (%)	0 (0.0)	63 (94)	
Alpha (B.1.1.7), n (%)	0 (0.0)	1 (1.5)	
Indeterminate, n (%)	0 (0.0)	3 (4.5)	
**Race[1], n (%)**			<0.001
White	795 (60)	201 (75)	
Black	94 (7)	8 (3)	
Mix-ethnicity (Pardo)	364 (28)	47 (18)	
Asian	15 (1)	3 (1)	
Not informed	47 (4)	9 (3)	
**Comorbidities, n (%)**			
Asthma	36 (3)	13 (5)	0.104
Cancer	129 (10)	10 (4)	0.002
Cardiovascular disease	194 (15)	54 (20)	0.034
Cerebrovascular disease	54 (4)	5 (2)	0.112
Chronic kidney disease	129 (10)	7 (3)	<0.001
Chronic pulmonary disease	77 (6)	17 (6)	0.868
Diabetes	501 (38)	79 (29)	0.009
Hypertension	751 (57)	122 (46)	0.001
Obesity	423 (35)	136 (51)	<0.001
HIV/AIDS	15 (1)	1 (0.4)	0.418

BMI: body mass index, kg/m2; IQR: interquartile range; SAPS 3: Simplified acute Physiology Score 3; SOFA: Sepsis-related Organ Failure Assessment. Data are presented as mean and standard deviation, unless otherwise stated; comparisons were made with t test, Mann–Whitney U test or Chi-square test as appropriate. Missing year 2020: BMI for 106 (8%) patients; SAPS3, missing for 1 patient. Missing

year 2021: BMI for 1 patient, SARS-CoV-2 variant screening for 201 (75%) patients in 2021
[1] The categories represent the Brazilian official race categories

Vaccination and variants of concern

In the second wave, nearly a third of patients were vaccinated with at least one dose of the COVID-19 vaccine. 67 (25%) patients in the second wave had samples available for SARS-CoV-2 mutations testing, 94% of whom were found to be infected with the Gamma variant (Table 1).

Ventilatory management on the first 24 hours after ICU admission

More patients required mechanical ventilation in the first 24 hours of their ICU stay in the first wave compared with the second (Table 2). The use of protective ventilation was common and similar in both waves. Rescue therapy for refractory hypoxemia was more often used in the second wave, mainly prone position, and PEEP titration, while respiratory system compliance and PaO_2/FiO_2 ratio were similar in both cohorts of patients.

Table 2. Ventilatory management on the first 24 h after ICU admission

Management	First wave (n=878)	Second wave (n=148)	*p* value
Tidal volume (mL/Kg ibw), mean (SD)	6.55 ± 1.3	6.17 ± 1.2	0.001
Respiratory Rate, median [IQR]	30 [26 - 35]	30 [25 - 31]	0.015
Minute volume, mean (SD)	12 ± 3.7	11 ± 2.7	<0.001
FiO2 (%), median [IQR]	50 [40 - 60]	50 [40 - 70]	0.178
PEEP (cmH2O), median [IQR]	10 [8 - 12]	10 [8 - 14]	<0.001
Plateau pressure (cmH2O), mean (SD)	22.6 ± 4.7	23.9 ± 4.3	0.001
Protective ventilation*, n (%)	638 (81)	130 (88)	0.076
Driving pressure (cmH2O), mean (SD)	12.6 ± 4	12.8 ± 3	0.686
Compliance (mLcmH2O−1), median [IQR]	32 [24 - 41]	30 [24 - 37]	0.070
Compliance (mLcmH2O−1.Kg−1 ibw), median [IQR]	0.52 [0.41 - 0.65]	0.48 [0.39 - 0.62]	0.022
PaO2/FIO2 (%), mean (SD)	168 ± 70	164 ± 72	0.521
Arterial pH, median [IQR]	7.36 [7.30 - 7.42]	7.36 [7.29 - 7.42]	0.986
Arterial PaCO2 (mmHg), median [IQR]	42.3 [37.9 - 48.3]	47.00 [39.0 - 56.1]	<0.001
Arterial O2 saturation (%), median [IQR]	93 [91 - 96]	94 [91 - 96]	0.687
**Ventilation Mode, n (%)**			<0.001
Volume-controlled ventilation	503 (57)	94 (64)	
Pressure-controlled ventilation	167 (19)	28 (19)	
Pressure support ventilation	199 (23)	15 (10)	
Other	9 (1)	11 (7)	
**Rescue therapy for hypoxemia, n (%)**			
Prone position	145 (16)	61 (41)	<0.001
PEEP titration	103 (12)	57 (38)	<0.001
Recruitment maneuvers	14 (1.6)	12 (8.1)	<0.001
Extracorporeal membrane oxygenation	1 (0.1)	2 (1.4)	0.079
Inhaled nitric oxide	1 (0.1)	0 (0.0)	-
**Protective ventilation, n (%)**	634 (82)	130 (88)	0.081

SD: standard deviation; IQR: interquartile range; O2: oxygen; PaCO2: arterial partial pressure of carbon dioxide; FIO2: inspired fraction of oxygen; PEEP: positive end-expiratory pressure; ibw: ideal body weight; PaO2/ FIO2: partial pressure of arterial oxygen and fraction of inspired oxygen ratio. Data are n. (%), unless otherwise stated; comparisons were made with t-test, Mann–Whitney U test or Chi-square test as appropriate Missing year 2020: Tidal volume (mL/Kg ibw) for 15 (1.7%) patients; PaO2/FIO2 was missing for 4 (0.5%) patients; plateau pressure (cmH2O) and driving pressure (cmH2O) were missing for 95 (11%) patients; PaCO2 for 58 (7%) patients; arterial pH for 52 (6%) patients and arterial O2 saturation for 61 (7%) patients. Missing year 2021: none

Management on the first 24 h after ICU admission

Differences in the clinical management of patients in the first 24 hours after ICU admission between waves are shown in S2 Table (Supporting information file). In the first wave, the percentage of patients under sedation was higher than in the second

wave, but use of neuromuscular blockade was more common on the second wave. The use of antibiotics was less common, and therapeutic anticoagulation was more frequently used in the second wave. Systemic corticosteroids were used for 98% of patients in the second wave, and only for 25% of patients in the first wave.

ICU and hospital outcomes

Relevant clinical outcomes are shown in Table 3. The median ICU stay in the first and second waves was not significantly different, but hospital stay was longer on the second wave. More patients in first wave required mechanical ventilation, while the use of NIV and/or HFNC were more frequent in the second wave, with similar duration of mechanical ventilation. Prone position was more commonly used in the second wave. Vasopressors and RRT were required more often in the first wave. The rate of most complications such as ventilator-associated pneumonia and cardiac arrhythmia was not different between waves, but thromboembolic events were more frequent in the second wave, and delirium was less frequent in the second wave.

Table 3. Clinical outcomes

Outcomes	First wave (n=1315)	Second wave (n=268)	*p* value
ICU length of stay, median [IQR], d	11 [6 - 19]	10 [7- 19]	0.445
Hospital length of stay, median [IQR], d	17 [11 - 27]	21 [14 - 32]	<0.001
Invasive mechanical ventilation, n (%)	1051 (80)	187 (70)	<0.001
Duration of mechanical ventilation, median [IQR], d	10 [6 - 17]	10 [6 - 19]	0.458
Reintubation, n (%)	206 (15.7)	17 (6.3)	<0.001
Prone positioning, n (%)	401 (38)	107 (57)	<0.001
Noninvasive ventilation[a], n (%)	291 (22)	123 (46)	<0.001
High-flow nasal cannula[a], n (%)	128 (10)	109 (41)	<0.001
Extracorporeal membrane oxygenation, n (%)	6 (0.5)	5 (1.9)	0.033
Vasopressors, n (%)	976 (74)	162 (60)	<0.001
Renal replacement therapy, n (%)	481 (37)	58 (22)	<0.001
Tracheostomy, n (%)	160 (12)	32 (12)	0.999
Delirium, n (%)	430 (33)	28 (10)	<0.001
Ventilator-associated pneumonia, n (%)	358 (27)	79 (29)	0.479
Thromboembolic event, n (%)	246 (19)	81 (30)	<0.001
Cardiac arrhythmia, n (%)	223 (17)	43 (16)	0.769
Treatment withhold or withdraw during hospital stay, n (%)	249 (19)	22 (8)	<0.001
Mortality at 28 days, n (%)	580 (44)	87 (32)	<0.001
Mortality at 60 days, n (%)	645 (49)	97 (36)	<0.001
**ICU outcome, n (%)**			<0.001
Discharged home	22 (1.7)	10 (3.7)	
Discharged to the ward	641 (49)	162 (60)	
Transferred to another ICU	50 (3.8)	6 (2.2)	
Transfer to long-term care facility	1 (0.1)	1 (0.4)	
Death	601 (46)	89 (33)	
**Hospital outcome, n (%)**			<0.001
Discharged home	553 (42)	162 (60)	
Transfer to long-term care facility	104 (8)	4 (1.5)	
Death	658 (50)	102 (38)	

ICU: intensive care unit; IQR: interquartile range; Data are n. (%), unless otherwise stated; comparisons were made with t test, Mann–Whitney U tests or chi-square test as appropriate
[a] To avoid intubation or prior to intubation

At the end of 60-days follow-up, 645 (49%) patients died in the first wave and 97 (36%) patients died in the second wave. After 60 days, another 13 (1%) patients in the first wave and 5 (2%) in the second wave died in the hospital.

Association of baseline characteristics with survival

Admission in the second wave was associated with higher survival at 60 days in the univariate analysis (HR 0.61, 95%CI 0.49 – 0.76), as shown in S3 Table (Supporting information file) and Fig 2A. After adjusting for age, SAPS 3 and vaccination, according to our conceptual causal diagram (Supporting information file: S1 Fig), admission in the second wave was no longer associated with survival (aHR 0.85, 95%CI 0.65–1.12). In this multivariable model, the only variables independently associated with 60-day hospital survival were age (aHR 1.02, 95%CI 1.02 – 1.03) and SAPS 3 (aHR 1.03, 95%CI 1.03 – 1.04), shown in S3 Table (Supporting information file) and Fig 2B.

Fig 2. Hospital 60-day survival according to year of admission.

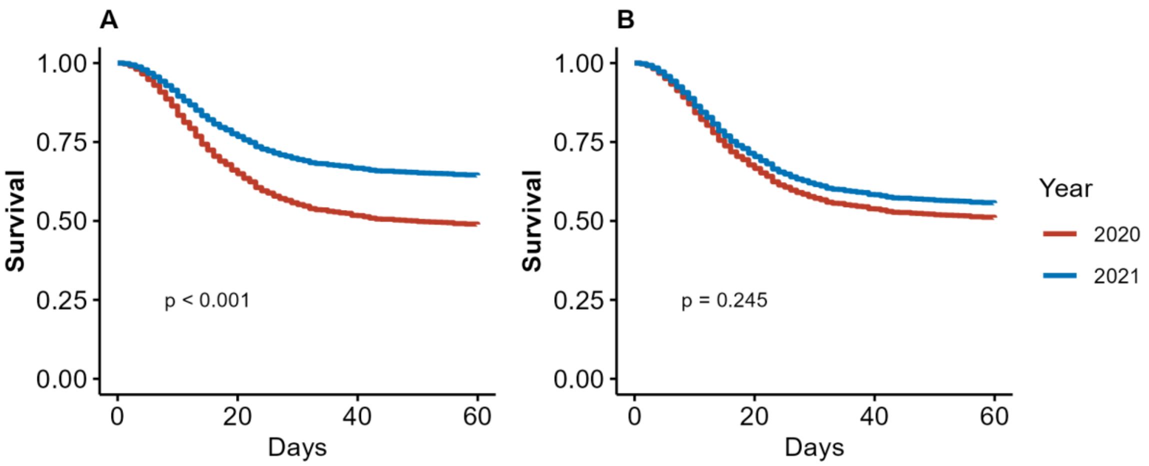

(A) Crude survival (HR 0.61, 95%CI 0.50 – 0.76). (B) Survival adjusted by age, SAPS 3 and vacination (aHR 0.85, 95%CI 0.65–1.12). Solid red line represents survival of patients who were admitted in 2020 (first wave) and solid blue line represents survival of patients who were admitted in 2021 (second wave). The *p* values were obtained with Cox proportional hazards models

Discussion

In this retrospective cohort, we compare characteristics and outcomes of 1,583 patients with COVID-19 admitted to the ICUs of a referral center for COVID-19 in Brazil in the first two waves of the pandemic. We found that 60-day mortality rate was 27% lower in the second wave compared to the first wave. However, in the multivariable analysis, adjusting by SAPS3, age and vaccination, admission to the ICU in the second wave was no longer associated with mortality. Patients admitted to the ICU in the second wave were younger, had better prognostic scores, and less need for vasopressors and RRT. Invasive mechanical ventilation was needed by most patients in both waves, 80% in the first and 70% in the second wave, and noninvasive ventilatory support and prone increased substantially in the second wave. Duration of mechanical ventilation and ICU length of stay were similar in the two waves, while hospital length of stay was longer in the second wave.

Survival at 60 days in our hospital was higher in the second wave than in the first wave, contrary to our study hypothesis, but in line with what was observed in studies from other countries [34–38]. Possible reasons for this difference include the admission of younger and less severe patients, earlier hospitalization, more organized and efficient ICU care, with better trained health care professionals, and more knowledge about the disease with better treatment protocols in the second wave. The impact of younger age and less severe disease at ICU admission on survival is supported by the finding that survival was similar in both waves when adjusted for age and SAPS3.

Our results contrast with the catastrophic outcomes seen in Brazil during the second wave, one the most affected countries in the world. By February 2021, the rapid spread of the Gamma variant was associated with a steep increase in number of cases [15], accompanied by a rise in the number of cases of SARI [39]. This combination of factors led to record hospitalizations and deaths [15] with signs of health system collapse [11,15,16], resulting in worse outcomes and potentially preventable deaths [40,41].

Access to ICU beds and availability of resources are very discrepant across Brazil, and such disparities increased during the pandemic [40]. Availability of ventilators was up to 5 times greater in southern and southeastern capitals compared to northern and northeastern capitals [40], and age-standardized mortality rates varied by up to 5 times between the northern and southern states in the second wave [39,42]. Our hospital is university-affiliated, located in the richest state of the country, with capability to increase ICU capacity and resources according to demand, and a centralized referral system which limited the number of patients admitted each day, in a striking contrast with frontline hospitals in the Amazon region, for example.

In Brazil and other countries, the second wave had an increased proportion of younger and previously healthy patients who required ICU care [15,34–36]. This reduction in the average age could be related to the fact that vaccination coverage started in January 2021 in Brazil and was associated with a reduction in hospitalizations and deaths in adults older than 70 years [26], but was not available to younger populations at that time. Another possible explanation to the high burden of cases among younger people in Brazil was a greater exposure to the virus during the second wave, when fewer restrictive measures were in effect, adherence to the use of masks decreased, and part of the economically active population had returned to work [28].

Patients in the second wave were less likely to need vasopressors and RRT during their ICU stay than patients in the first wave, as noted in other studies [34,35,43]. These results support the finding that better clinical outcomes in the second wave could be associated with younger age and less severe disease at ICU admission.

During the second wave, we found that 94% of the samples analyzed detected the Gamma variant, in accordance to epidemiological findings in Brazil during this period [5]. The Gamma variant spread throughout Brazil and other countries and was found to be more transmissible [4–6], having a higher risk of hospitalization [39,44]. While some

studies found that it caused more severe disease and increased the risk of death [39,41,45], others did not see an increased mortality [43,46,47]. It is possible that the elevated daily death counts observed during the second wave in Brazil was due to a combination of increased risk of development of SARI [39] and lack or delayed access to healthcare and strain on the healthcare system.

Management of patients in the second wave was influenced by accumulated knowledge about the efficacy of several therapeutic options. Importantly, less use of antibiotics [48], and more use of corticosteroids were informed by randomized controlled trials [49,50]. In our cohort, only 25% of the patients received corticosteroid in the first wave, while 98% received it in the second wave, which may have had an impact in survival.

The use of noninvasive ventilatory support increased during the second wave, likely due to greater availability, more experience, and less concern about and environmental contamination [51]. Use of noninvasive support may have contributed to avoiding intubations in the second wave, in which we found 10% less use of invasive mechanical ventilation compared to the first wave. Currently, there is evidence that noninvasive ventilatory support is effective in decreasing intubation rates, but not mortality, compared to conventional oxygen in COVID-19 [52,53].

Among intubated patients, we found that the more than 80% of patients received protective ventilation in both waves, which our group previously found to be associated with increased survival in COVID-19 [2]. Rescue therapies, including recruiting maneuvers, PEEP titration, and extracorporeal membrane oxygenation were more often used in the second wave. The same observation was seen with prone position, which was used in 57% of the patients in the second wave, compared to 38% in the first wave. Prone position was increasingly used during the COVID-19 pandemic, probably based on better training of health professionals, greater knowledge in the

management of patients with COVID-19 pneumonia, and previous evidence of benefit of prone in patients with moderate to severe non-COIVD-19 ARDS [54].

Our study has several limitations. Being a retrospective observational study, data were collected from the electronic medical record, which could have incomplete or inaccurate data. It is a single-center study, and our findings may not be generalizable to other hospitals in Brazil and other low-and-middle-income countries. However, it is our belief that this does not have a significant impact on the inferential conclusions related to the comparison between COVID-19 waves. Only 25% of respiratory samples from patients in the second wave were available for variant testing, because many patients were diagnosed in other health services before being transferred to our hospital or diagnosed with antigen tests, limiting our power to estimate the association of variants of concern with clinical outcomes. We compared the first two waves of COVID-19 in Brazil, which happened in different contexts of hospital organization. During the first wave, our hospital had five times more ICU beds compared to the second wave, reflecting greater strain. Our study was unable to measure the impact of updated treatment protocols, better structuring of ICUs and training of health care professionals in our hospital that occurred in the second wave. The sample of patients admitted in the second wave was smaller, which limited our statistical power. Finally, since our study only included ICU patients, we could not measure the impact of the Gamma variant on increased risk of development of SARI, which in turn may have been responsible for overall increased risk of death in the second wave.

Our study also has strengths. The hospital covers a large geographical area, with an estimated population of 23 million; we screened all consecutive patients with confirmed COVID-19 admitted to the participating ICUs, minimizing selection bias; we included a large sample size; we had complete follow-up of patients until hospital discharge or transfer to another health service; we had few missing data; and finally, we assessed objective, hard outcomes.

Conclusion

In this cohort study conducted across multiple ICUs of the largest academic hospital in Brazil, we observed that patients admitted to the ICU in the second wave of the COVID-19 pandemic were younger and had better prognostic scores compared to those in the first wave. They also required fewer advanced life support therapies and had higher survival rates. Survival rates adjusted for age and severity score were found to be similar between waves. These results contrast with the country-level outcomes and underscore the impact of disease severity at ICU admission, as well as the availability and rational allocation of healthcare resources, health-system strain, and health disparities on the outcome of the pandemic.

Abbreviations

aHR	Adjusted hazard ratio
ARDS	Acute Respiratory Distress Syndrome
BMI	Body mass index
COVID-19	Coronavirus Disease 2019
DAG	Directed acyclic graph
FiO2	Fraction of inspired oxygen
HCFMUSP	Hospital das Clínicas da Faculdade de Medicina da Universidade de São Paulo
HFNC	High-flow nasal cannula
HR	Hazard ratio
IBW	Ideal body weight
ICU	Intensive care unit
IgG	Immunoglobulin G
IgM	Immunoglobulin M
IQR	Interquartile range
NIV	Noninvasive ventilation
PaO2/FiO2	Partial pressure of arterial oxygen and fraction of inspired oxygen ratio
PEEP	Positive end-expiratory pressure
REDCap	Research Electronic Data Capture
RRT	Renal replacement therapy

RT-PCR	Reverse Transcription Polymerase Chain Reaction
SAPS 3	Simplified Acute Physiology Score III
SARI	Severe acute respiratory infection
SARS-CoV-2	Severe Acute Respiratory Syndrome - Coronavirus 2
SD	standard deviation
SOFA	Sequential (Sepsis-related) Organ Failure Assessment
STROBE	Strengthening The Reporting of Observational Studies in Epidemiology
VOCs	Variants of concern

Acknowledgements

We would like to thank the Hospital das Clinicas COVID-19 crisis committee, healthcare workers and staff for their important work at our hospital during the COVID-19 pandemic.

References

1. World Health Organization (WHO). Coronavirus Disease (COVID-19) Dashboard. Geneva: WHO; 2023. https://covid19.who.int/. Accessed 7 July 2023.

2. Ferreira JC, Ho YL, Besen BAMP, Malbouisson LMS, Taniguchi LU, Mendes PV, et al; EPICCoV Study Group. Protective ventilation and outcomes of critically ill patients with COVID-19: a cohort study. Ann Intensive Care. 2021 Jun 7;11(1):92.

3. Ranzani OT, Bastos LSL, Gelli JGM, Marchesi JF, Baião F, Hamacher S, et al. Characterisation of the first 250 000 hospital admissions for COVID-19 in Brazil: a retrospective analysis of nationwide data. Lancet Respir Med. 2021 Apr 1;9(4):407–418.

4. World Health Organization (WHO). Tracking SARS-CoV-2 variants. Geneva: WHO; 2023. https://www.who.int/activities/tracking-SARS-CoV-2-variants. Accessed 24 Oct

2022

15. Gräf T, Bello G, Naveca FG, Gomes M, Cardoso VLO, da Silva AF, et al. Phylogenetic-based inference reveals distinct transmission dynamics of SARS-CoV-2 lineages Gamma and P.2 in Brazil. iScience. 2022 Apr 15;25(4):104156.

16. Faria NR, Mellan TA, Whittaker C, Claro IM, Candido DDS, Mishra S, et al. Genomics and epidemiology of the P.1 SARS-CoV-2 lineage in Manaus, Brazil. Science. 2021 May 21;372(6544):815-821.

17. Hoffmann M, Arora P, Groß R, Seidel A, Hörnich BF, Hahn AS, et al. SARS-CoV-2 variants B.1.351 and P.1 escape from neutralizing antibodies. Cell. 2021 Apr 29;184(9):2384-2393.e12.

18. Souza WM, Amorim MR, Sesti-Costa R, Coimbra LD, Brunetti NS, Toledo-Teixeira DA, et al. Neutralisation of SARS-CoV-2 lineage P.1 by antibodies elicited through natural SARS-CoV-2 infection or vaccination with an inactivated SARS-CoV-2 vaccine: an immunological study. Lancet Microbe. 2021 Oct;2(10):e527–e535.

19. Harvey WT, Carabelli AM, Jackson B, Gupta RK, Thomson EC, Harrison EM, et al. SARS-CoV-2 variants, spike mutations and immune escape. Nat Rev Microbiol. 2021 Jul;19(7):409-424.

20. Hahn G, Wu CM, Lee S, Lutz SM, Khurana S, Baden LR, et al. Genome-wide association analysis of COVID-19 mortality risk in SARS-CoV-2 genomes identifies mutation in the SARS-CoV-2 spike protein that colocalizes with P.1 of the Brazilian strain. Genet Epidemiol. 2021 Oct;45(7):685–693.

21. Silva SJR, Pena L. Collapse of the public health system and the emergence of new variants during the second wave of the COVID-19 pandemic in Brazil. One Health. 2021 Dec;13:100287.

42. Lin L, Liu Y, Tang X, He D. The Disease Severity and Clinical Outcomes of the SARS-CoV-2 Variants of Concern. Front Public Health. 2021 Nov 30;9:775224.

43. Nicolete VC, Rodrigues PT, Fernandes ARJ, Corder RM, Tonini J, Buss LF, et al. Epidemiology of COVID-19 after Emergence of SARS-CoV-2 Gamma Variant, Brazilian Amazon, 2020-2021. Emerg Infect Dis. 2022 Mar;28(3):709–712.

44. Fundação Oswaldo Cruz (Brasil). A Rede Genômica. Rio de Janeiro: FIOCRUZ, 2022. Portuguese. http://www.genomahcov.fiocruz.br/. Accessed 26 Oct 2022.

45. Bastos LS, Ranzani OT, Souza TML, Hamacher S, Bozza FA. COVID-19 hospital admissions: Brazil's first and second waves compared. Lancet Respir Med. 2021 Aug;9(8):e82-e83.

46. Fundação Oswaldo Cruz (Brasil). COVID-19 Observatory points to the biggest health system collapse in the history of Brazil. Rio de Janeiro: FIOCRUZ, 2022. https://portal.fiocruz.br/en/news/covid-19-observatory-points-biggest-health-system-collapse-history-brazil. Accessed 26 Oct 2022.

47. Lobo SM, Mello PMVC. Challenges of the coronavirus pandemic for Brazilian intensivists: present and future. Rev Bras Ter Intensiva. 2021 Sep 20:S0103-507X2021005001203. Portuguese, English.

48. Challen R, Brooks-Pollock E, Read JM, Dyson L, Tsaneva-Atanasova K, Danon L. Risk of mortality in patients infected with SARS-CoV-2 variant of concern 202012/1: Matched cohort study. BMJ. 2021 Mar 9;372:n579.

49. Salyer SJ, Maeda J, Sembuche S, Kebede Y, Tshangela A, Moussif M, et al. The first and second waves of the COVID-19 pandemic in Africa: a cross-sectional study. Lancet. 2021 Apr 3;397(10281):1265–1275.

20. Davies NG, Jarvis CI, CMMID COVID-19 Working Group, Edmunds WJ, Jewell NP, Diaz-Ordaz K, et al. Increased mortality in community-tested cases of SARS-CoV-2 lineage B.1.1.7. Nature. 2021 May;593(7858):270-274.

21. Abdool Karim SS, de Oliveira T. New SARS-CoV-2 Variants - Clinical, Public Health, and Vaccine Implications. N Engl J Med. 2021 May 13;384(19):1866-1868.

22. Whittaker C, Ratmann O, Dye C, Sabino EC, Faria NR. Altered demographic profile of hospitalizations during the second COVID-19 wave in Amazonas, Brazil. Brazil. Lancet Reg Health Am. 2021 Oct;2:100064.

23. Freitas ARR, Beckedorff OA, Cavalcanti LPG, Siqueira AM, Castro DB, Costa CFD, et al. The emergence of novel SARS-CoV-2 variant P.1 in Amazonas (Brazil) was temporally associated with a change in the age and sex profile of COVID-19 mortality: A population based ecological study. Lancet Reg Health Am. 2021 Sep; 1:100021.

24. Spira B. The Impact of the Highly Virulent SARS-CoV-2 Gamma Variant on Young Adults in the State of São Paulo: Was It Inevitable? Cureus. 2022 Jul 1;14(7):e26486.

25. de Souza FSH, Hojo-Souza NS, de Oliveira DCR, Silva CMD, Guidoni DL. An overview of Brazilian working age adults vulnerability to COVID-19. Sci Rep. 2022 Feb 18;12(1):2798.

26. Ranzani OT, Hitchings MDT, Dorion M, D'Agostini TL, de Paula RC, de Paula OFP, et al. Effectiveness of the CoronaVac vaccine in older adults during a gamma variant associated epidemic of covid-19 in Brazil: test negative case-control study. BMJ. 2021 Sep 6;374:n2091. Erratum for: BMJ. 2021 Aug 20;374:n2015.

27. BRASIL. Ministério da Saúde. Secretaria de Vigilância em Saúde. Departamento de Imunização e Doenças Transmissíveis. Plano Nacional de Operaracionalização da Vacinação contra a COVID-19 [National Plan for the Operationalization of Vaccination

against COVID-19]. 7th ed. p. 1–108. Brasília, 2021. Portuguese. https://sbim.org.br/images/files/notas-tecnicas/plano-nacional-operacionalizacao-planovacinacaocovid-ed7-20210517-cgpni.pdf. Acessed 4 Jan 2023.

28. Hallal PC, Victora CG. Overcoming Brazil's monumental COVID-19 failure: an urgent call to action. Nat Med. 2021 Jun;27(6):933.

29. Harris PA, Taylor R, Minor BL, Elliott V, Fernandez M, O'Neal L, et al. The REDCap consortium: Building an international community of software platform partners. J Biomed Inform. 2019 Jul;95:103208.

30. Moreno RP, Metnitz PG, Almeida E, Jordan B, Bauer P, Campos RA, et al; SAPS 3 Investigators. SAPS 3--From evaluation of the patient to evaluation of the intensive care unit. Part 2: Development of a prognostic model for hospital mortality at ICU admission. Intensive Care Med. 2005 Oct;31(10):1345-55. Erratum in: Intensive Care Med. 2006 May;32(5):796.

31. Vincent JL, Moreno R, Takala J, Willatts S, de Mendonça A, Bruining H, et al. The SOFA (Sepsis-related Organ Failure Assessment) score to describe organ dysfunction/failure. On behalf of the Working Group on Sepsis-Related Problems of the European Society of Intensive Care Medicine. Intensive Care Med. 1996 Jul;22(7):707-10.

32. von Elm E, Altman DG, Egger M, Pocock SJ, Gøtzsche PC, Vandenbroucke JP; STROBE Initiative. The Strengthening the Reporting of Observational Studies in Epidemiology (STROBE) Statement: guidelines for reporting observational studies. Int J Surg. 2014 Dec;12(12):1495-9.

33. R Core Team (2022). R: A Language and Environment for Statistical Computing. R Foundation for Statistical Computing, Vienna, Austria, 2022.

https://posit.cloud

34. Hoogenboom WS, Pham A, Anand H, Fleysher R, Buczek A, Soby S, et al. Clinical characteristics of the first and second COVID-19 waves in the Bronx, New York: A retrospective cohort study. Lancet Reg Health Am. 2021 Nov;3:100041.

35. Dongelmans DA, Termorshuizen F, Brinkman S, Bakhshi-Raiez F, Arbous MS, de Lange DW, et al. Characteristics and outcome of COVID-19 patients admitted to the ICU: a nationwide cohort study on the comparison between the first and the consecutive upsurges of the second wave of the COVID-19 pandemic in the Netherlands. Ann Intensive Care. 2022 Jan 13;12(1):5.

36. Domingo P, Pomar V, Mur I, Castellví I, Corominas H, de Benito N. Not all COVID-19 pandemic waves are alike. Clin Microbiol Infect. 2021 Jul;27(7):1040.e7-1040.e10.

37. Fan G, Yang Z, Lin Q, Zhao S, Yang L, He D. Decreased Case Fatality Rate of COVID-19 in the Second Wave: A study in 53 countries or regions. Transbound Emerg Dis. 2021 Mar;68(2):213-215.

38. Iftimie S, Lopez-Azcona AF, Vallverdu I, Hernandez-Flix S, de Febrer G, Parra S, et al. First and second waves of coronavirus disease-19: A comparative study in hospitalized patients in Reus, Spain. PLoS One. 2021 Mar 31;16(3):e0248029.

39. Brizzi A, Whittaker C, Servo LMS, Hawryluk I, Prete CA Jr, de Souza WM, et al. Spatial and temporal fluctuations in COVID-19 fatality rates in Brazilian hospitals. Nat Med. 2022 Jul;28(7):1476-1485.

40. Silva GAE, Jardim BC, Lotufo PA. Mortalidade por COVID-19 padronizada por idade nas capitais das diferentes regiões do Brasil [Age-adjusted COVID-19 mortality in state capitals in different regions of Brazil]. Cad Saude Publica. 2021 Jul 7;37(6):e00039221. Portuguese.

41. Prete CA, Buss LF, Whittaker C, Salomon T, Oikawa MK, Pereira RHM, et al. SARS-CoV-2 antibody dynamics in blood donors and COVID-19 epidemiology in eight Brazilian state capitals: A serial cross-sectional study. Elife. 2022 Sep 22;11:e78233.

42. Zeiser FA, Donida B, da Costa CA, Ramos GO, Scherer JN, Barcellos NT, et al. First and second COVID-19 waves in Brazil: A cross-sectional study of patients' characteristics related to hospitalization and in-hospital mortality. Lancet Reg Health Am. 2022 Feb;6:100107.

43. Lázaro APP, Albuquerque PLMM, Meneses GC, Zaranza MS, Batista AB, Aragão NL, et al. Critically ill COVID-19 patients in northeast Brazil: mortality predictors during the first and second waves including SAPS 3. Trans R Soc Trop Med Hyg. 2022 Nov 1;116(11):1054-1062.

44. Paredes MI, Lunn SM, Famulare M, Frisbie LA, Painter I, Burstein R, et al. Associations Between Severe Acute Respiratory Syndrome Coronavirus 2 (SARS-CoV-2) Variants and Risk of Coronavirus Disease 2019 (COVID-19) Hospitalization Among Confirmed Cases in Washington State: A Retrospective Cohort Study. Clin Infect Dis. 2022 Aug 24;75(1):e536-e544.

45. Nonaka CKV, Gräf T, Barcia CAL, Costa VF, de Oliveira JL, Passos RDH, et al. SARS-CoV-2 variant of concern P.1 (Gamma) infection in young and middle-aged patients admitted to the intensive care units of a single hospital in Salvador, Northeast Brazil, February 2021. Int J Infect Dis. 2021 Oct;111:47-54.

46. Oliveira EA, Simões E Silva AC, Oliveira MCL, Colosimo EA, Mak RH, Vasconcelos MA, et al. Comparison of the First and Second Waves of the Coronavirus Disease 2019 Pandemic in Children and Adolescents in a Middle-Income Country: Clinical Impact Associated with Severe Acute Respiratory Syndrome Coronavirus 2 Gamma Lineage. J Pediatr. 2022 May;244:178-185.e3.

47. Santos RS, Barros DS, Moraes TMP, Hayashi CY, Ralio RB, Minenelli FF, et al. Clinical characteristics and outcomes of hospitalized patients with COVID-19 in a Brazilian hospital: a retrospective study of the first and second waves. IJID Regions. 2022 Jun;3:189–195.

48. Cavalcanti AB, Zampieri FG, Rosa RG, Azevedo LCP, Veiga VC, Avezum A, et al. Hydroxychloroquine with or without Azithromycin in Mild-to-Moderate Covid-19. N Engl J Med. 2020 Nov 19;383(21):2041-2052. Erratum in: N Engl J Med. 2020 Nov 19;383(21):e119.

49. RECOVERY Collaborative Group; Horby P, Lim WS, Emberson JR, Mafham M, Bell JL, Linsell L, et al. Dexamethasone in Hospitalized Patients with Covid-19. N Engl J Med. 2021 Feb 25;384(8):693-704.

50. Tomazini BM, Maia IS, Cavalcanti AB, Berwanger O, Rosa RG, Veiga VC, et al. Effect of Dexamethasone on Days Alive and Ventilator-Free in Patients with Moderate or Severe Acute Respiratory Distress Syndrome and COVID-19: The CoDEX Randomized Clinical Trial. JAMA. 2020 Oct 6;324(13):1307-1316.

51. Winslow RL, Zhou J, Windle EF, Nur I, Lall R, Ji C, et al. SARS-CoV-2 environmental contamination from hospitalised patients with COVID-19 receiving aerosol-generating procedures. Thorax. 2022 Mar;77(3):259–267.

52. Perkins GD, Ji C, Connolly BA, Couper K, Lall R, Baillie JK, et al. Effect of Noninvasive Respiratory Strategies on Intubation or Mortality among Patients with Acute Hypoxemic Respiratory Failure and COVID-19: The RECOVERY-RS Randomized Clinical Trial. JAMA. 2022 Feb 8;327(6):546–558.

53. Frat JP, Quenot JP, Badie J, Coudroy R, Guitton C, Ehrmann S, et al. Effect of High-Flow Nasal Cannula Oxygen vs Standard Oxygen Therapy on Mortality in Patients with

Respiratory Failure Due to COVID-19: The SOHO-COVID Randomized Clinical Trial. JAMA. 2022 Sep 27;328(12):1212-1222.

34. Guérin C, Reignier J, Richard JC, Beuret P, Gacouin A, Boulain T, et al; PROSEVA Study Group. Prone positioning in severe acute respiratory distress syndrome. N Engl J Med. 2013 Jun 6;368(23):2159-68.

 Click here to access/download;Figure;Fig 1.tif

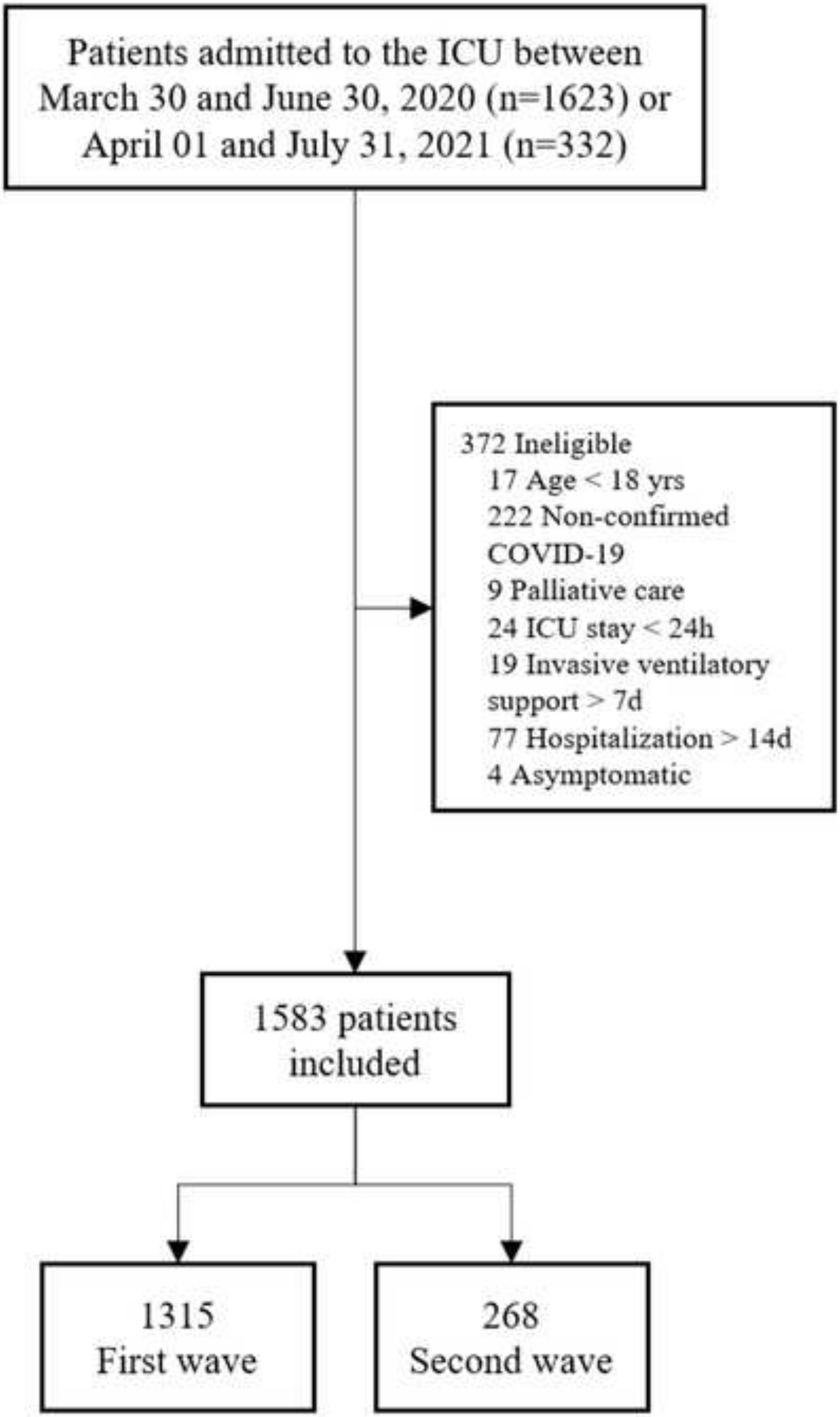

 Click here to access/download;Figure;Fig 2.tif

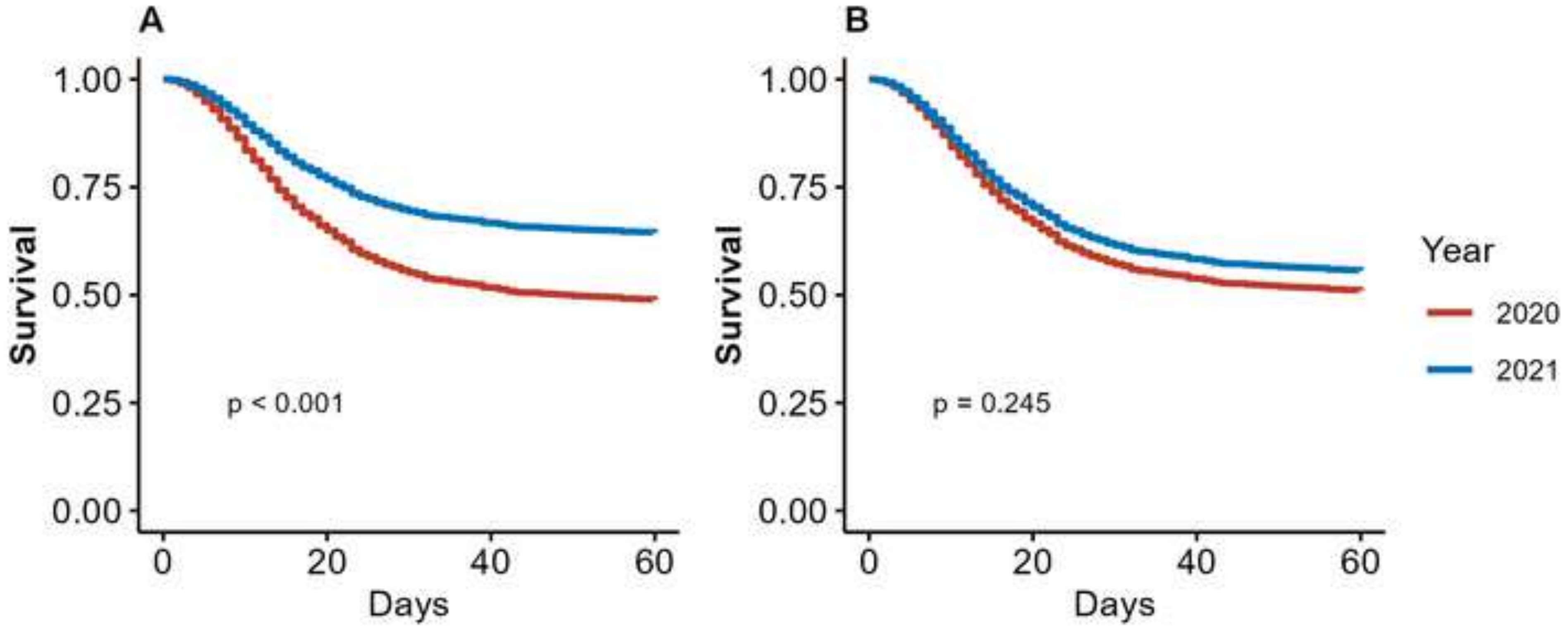

Click here to access/download;Figure;FigureS1.tif

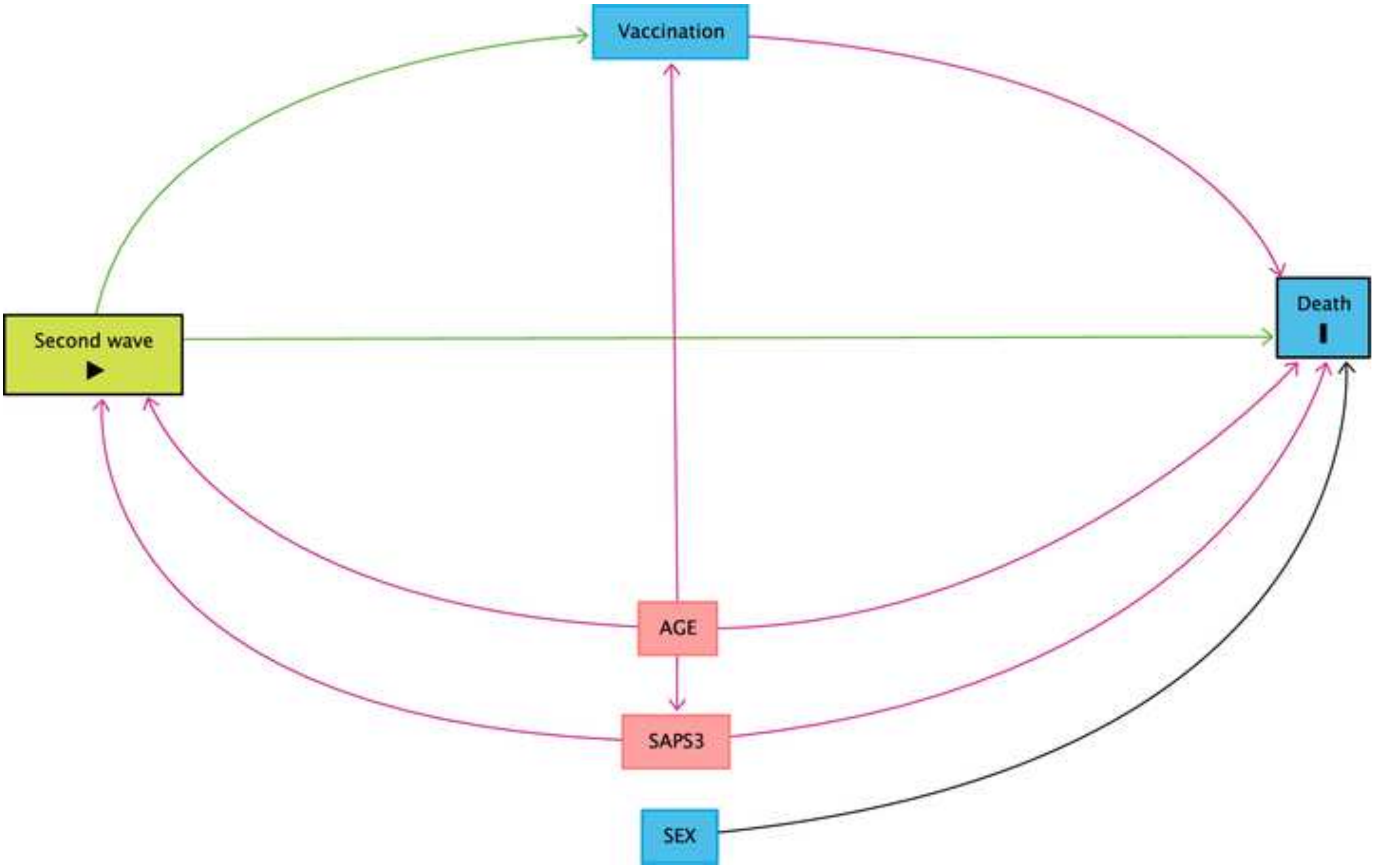

Click here to access/download
Supporting Information
Supporting information file.docx
